# *N*-Glycosylation of integrin α5 acts as a switch for EGFR-mediated complex formation of integrin α5β1 to α6β4

**DOI:** 10.1038/srep33507

**Published:** 2016-09-19

**Authors:** Qinglei Hang, Tomoya Isaji, Sicong Hou, Ying Zhou, Tomohiko Fukuda, Jianguo Gu

**Affiliations:** 1 Division of Regulatory Glycobiology, Institute of Molecular Biomembrane and Glycobiology, Tohoku Medical and Pharmaceutical University, 4-4-1 Komatsushima, Aoba-ku, Sendai, Miyagi, 981-8558, Japan

## Abstract

*N*-Glycosylation of integrin α5β1 is involved in multiple cell behaviors. We previously reported that the *N*-glycosylations of the calf domain on integrin α5 (S3–5,10–14) are essential for its inhibitory effect on EGFR signaling in regulating cell proliferation. However, the importance of the individual *N*-glycosylation and the underlying mechanisms of inhibition remain unclear. Here, we characterize the S3–5,10–14 mutants in detail and found that the *N*-glycosylation of site-11 (Asn712) is key for cell growth. The restoration of site-11, unlike the other individual sites, significantly suppressed cell growth and EGFR signaling in a manner that was similar to that of wild-type (WT). Mechanistically, this *N*-glycosylation inhibited the response abilities upon EGF stimulation and EGFR dimerization. Interestingly, we found this *N*-glycosylation controlled the EGFR complex formation with integrin α5β1 or α6β4; i.e., the loss of site-11 switched EGFR-α5β1 to EGFR-α6β4, which is well known to promote cellular signaling for cell growth. Moreover, the site-11 *N*-glycan exhibited a more branching structure compared with other sites, which may be required for EGFR-α5β1 formation. Taken together, these data clearly demonstrate that the site-11 *N*-glycosylation on α5 is most important for its inhibitory effect on EGFR signaling, which may provide a novel regulatory mechanism for crosstalks between integrins and EGFR.

Integrins are αβ heterodimeric transmembrane glycoproteins that link cells to their surroundings such as to the cell-extracellular matrix (ECM) or to other cells^1^. In addition to performing a structural role, integrins bidirectionally relay signals, outside-in and inside-out, across the cell membrane^2^,^3^, and participate in the growth factor receptors (GFRs) signaling pathways^4^,^5^. The crosstalk between epidermal growth factor receptor (EGFR), a well-known GFR, and integrins plays a key role in multiple biological processes in tumors, including cell growth, metastasis, and drug resistance^6^. However, the effect that integrins exert on the regulation of EGFR-mediated signaling seems rather indiscriminate, since a variety of integrins, such as integrin α5β1, α6β4, αvβ3, and αvβ5^7^,^8^,^9^,^10^, have been respectively reported to regulate EGFR signaling, and also the effects of several integrins on EGFR signaling or cell growth are controversial, such as integrin α5β1^11^,^12^,^13^ and α6β4^14^,^15^, which prompted us to explore how integrins regulate EGFR activation in a coordinated manner.

Recently, an accumulating body of evidence has begun revealing mechanisms that describe how integrins and GFRs collaborate at many different levels from gene to post-transcriptional regulation^16^. For instance, the mechanisms involved in how integrins regulate EGFR signaling seem to most commonly converge on kinases such as Src family kinases (SFKs)^17^, scaffolding proteins such as p130 Crk-associated substrates (p130^Cas^)^18^, GTPases-related proteins such as the Ras and Rab family^16^, and phosphatases such as T-cell protein tyrosine phosphatase^19^, which drives largely around the inner membrane. However, these findings are insufficient to clarify the molecular mechanisms in detail.

In contrast, knowledge is limited regarding the regulatory mechanisms of the outer membrane crosstalks mediated by integrins. In recent years, *N*-glycosylation was believed to be important in this respect, given its roles in regulating cellular signalings, as it provides a set of attractive targets for the diagnosis and treatment of cancer^20^,^21^. Several research groups, including ours, have demonstrated that alterations of the *N*-glycan composition on several integrins could regulate their association with other membrane proteins, such as EGFR, urokinase-type plasminogen activation receptor, and tetraspanin proteins^22^,^23^,^24^. In addition, we found that the *N*-glycosylation of a specific domain in the integrin α5 subunit could regulate EGFR signaling and cell proliferation^11^. Nevertheless, further investigation is needed to fully explore the molecular mechanisms underlying this regulation.

The *N*-glycosylation of integrin α5β1 appears to be very important for its mediation of multiple biological functions including heterodimer formation, cell surface expression, ligand binding, cell adhesion, and migration^25^. Indeed, complete ablation of *N*-glycosylation on integrin α5β1 by treatment with tunicamycin and *N*-glycosidase F prevents both transport to the cell surface and binding to fibronectin (FN), respectively^26^,^27^. Also, changes in *N*-glycan composition of integrin α5β1 could regulate its binding ability to FN and its cell spreading and migration via the overexpression of glycosyltransferase genes such as *N*-acetylglucosaminyltransferase-V (GnT-V), GnT-III, or α2,6-galactoside sialyltransferase 1 (ST6GAL1)^28^,^29^,^30^,^31^. However, most of the previous studies have examined only total changes by displaying or masking specific glycan epitopes without individual information. In fact, integrin α5β1 is a major carrier of *N*-glycans, particularly the α5 portion that contains 14 potential *N*-glycosylation sites. This complexity raises important questions as to whether individual *N*-glycosylation of α5 plays a specific role in modulating its function. Our previous study revealed that *N*-glycosylation sites 3–5, and site-5 in particular, on the β-propeller domain of α5 is essential for the assembly of α5β1 heterodimer formation and its expression on the cell surface^32^, which suggests that individual *N*-glycosylation has its own function.

Given the importance of the *N*-glycosylation of integrins in EGFR activation, we considered there might be one or several specific *N*-glycosylation(s) of α5 that regulate EGFR signaling for the control of cell proliferation. To test this hypothesis, we characterized the *N*-glycosylation of the calf domain in α5 in detail and found that *N*-glycosylation of site-11 plays a key role in its inhibitory effect on EGFR function. Furthermore, we found that the site-11 *N*-glycosylation at least partially participates in switching the EGFR complex formation from integrin α5β1 to α6β4. In addition, the specific structures of site-11 N-glycan may also be important for the functions, since the site-11 N-glycan exhibited more branching structures and the suppression of these branching structures by overexpression of GnT-III significantly decreased the EGFR-integrins complex formation. These findings may provide new insight into the cellular signaling machinery.

## Results

### The potential *N*-glycosylation of site-11 on the calf domain of integrin α5 is the most important aspect in the development of its inhibitory function

**Figure 1:**
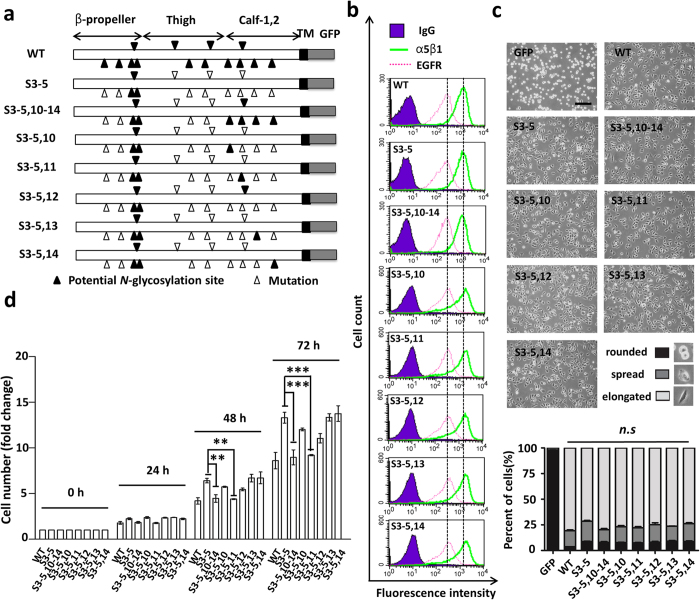
Effects of various unglycosylated mutant α5 subunits expression on cell growth in CHO-B2/EGFR cells. (**a**) Schematic diagram of potential *N*-glycosylation sites on the WT and mutational integrin α5 subunits (WT; S3–5; S3–5,10–14; S3–5,10; S3–5,11; S3–5,12; S3–5,13 and, S3–5,14). Putative *N*-glycosylation sites (N84Q, N182Q, N297Q, N307Q, N316Q, N524Q, N530Q, N593Q, N609Q, N675Q, N712Q, N724Q, N773Q, and N868Q) are indicated by solid triangles, and point mutations are indicated by hollow triangles. (**b**) The integrin α5 mutant cells expressed equal α5β1 and EGFR levels on the cell surface, compared with the WT cells. The stable cell lines were established as described under the “Methods” section. The expression levels of both α5β1 and EGFR were analyzed by flow cytometry. The IgG was used as a control. (**c**) The α5 mutant cells exhibited abilities for cell spreading that were comparable to those of WT. Cells were detached and then replated on the FN-coated dishes. After incubation for 20 min, cells were fixed and the representative images were taken. The percentages of rounded, spread, and elongated cells were statistically analyzed (*right bottom panel, n* = 9). *Scale bar*, 120 μm. (**d**) The S3–5,11 cells exhibited inhibitory ability for cell growth that was comparable to that of the S3–5,10–14 versions. After starvation for 24 h, cells were supplied with DMEM containing 10% FBS, and then cell numbers were counted and statistically analyzed at indicated times (*n* = 3 individual experiments). All values are reported as the means ± S.E. (*error bars*), Student’s *t*-test; n.s, not significant (*p* > *0.05*); **p < 0.01; ***p < 0.001.

In prior work we identified three *N*-glycosylation sites on the β-propeller domain (S3–5) (Fig. 1a) of the integrin α5 subunit that are essential for α5β1 heterodimerization, cell surface expression, and cell adhesion in CHO-B2 cells^32^. Based on this, we recently found that *N*-glycosylation of the calf domain, which localizes in the vicinity of the cell membrane of integrin α5 (S3–5,10–14) (Fig. 1a), plays a key role in its inhibitory effect on EGFR signaling^11^. These findings led to the question of whether there is/are one or several specific *N*-glycosylation(s) of α5 that is needed to regulate the crosstalk between integrin α5 and EGFR.

To test this concept, using the form with *N*-glycosylation sites 3–5 reintroduced, we replaced one-by-one the sites 10–14 within the calf domain, as shown in Fig. 1a (S3–5,10; S3–5,11; S3–5,12; S3–5,13; and, S3–5,14), in the CHO-B2/EGFR cells, which is an integrin α5 deficient but stable overexpression of EGFR cell line we established previously^11^. As shown in Fig. 1b, in flow cytometry analysis these mutant cells exhibited almost the same expression levels of EGFR and α5β1 on the cell surface by comparison with WT, which suggested that individual *N*-glycosylation of the calf domain has no effect on the expression of EGFR. Meanwhile, these mutants exhibited abilities for cell spreading on fibronectin (FN) that were comparable to those of WT cells (Fig. 1c), which also indicated that these five individual *N*-glycosylation had no significant effect on cell spreading. These results suggest that these re-expressed individual *N*-glycosylation mutants may serve as a reliable cell model that could be used to compare their effects on EGFR signaling and how it regulates cell proliferation.

Next, we compared the cell proliferation ability among these five mutants. As shown in Fig. 1d, cell proliferation was partially decreased in the S3–5,10; S3–5,11; and, S3–5,12 cells, but not in the other two mutants (S3–5,13 and S3–5,14), compared with that in S3–5 cells. However, only S3–5,11 mutants showed significant inhibitory effects on cell proliferation, as the S3–5,10–14 or the WT had. These results suggest that the function of WT integrin α5 on cell proliferation can be duplicated by the S3–5,11 mutant, and that the *N*-glycosylation of site-11 is the most important development for its inhibitory functions.

### EGFR-related cellular signaling is strictly controlled by the site-11 *N*-glycosylation of integrin α5

**Figure 2:**
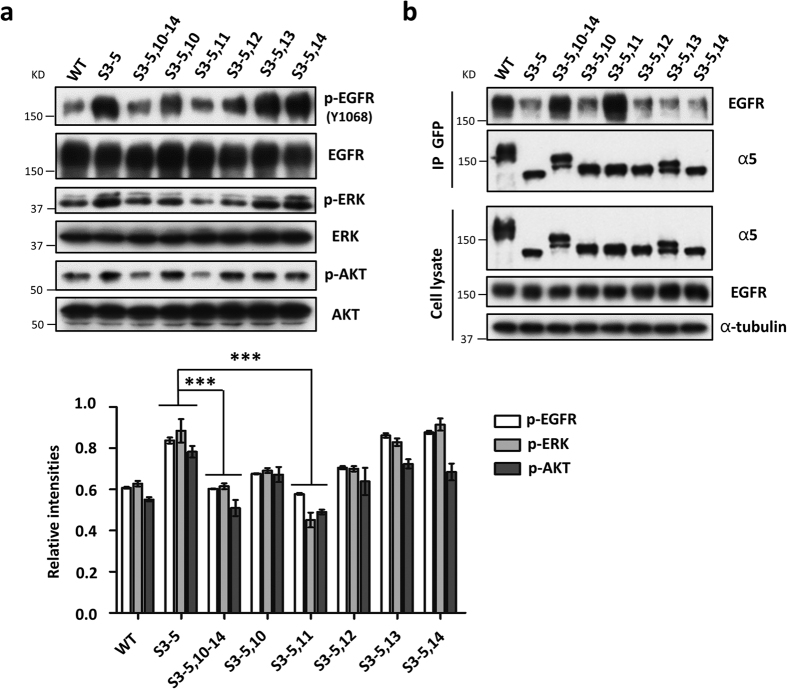
Comparison of the levels of phosphorylated EGFR, related cellular signaling, and α5-EGFR complex formation among various unglycosylated α5 mutants in CHO-B2/EGFR cells. (**a**) The expression levels of phospho-EGFR and its downstream signaling were down-regulated in the S3–5,11 cells. Cell lysates from the indicated cells were subjected to western blot (*WB*) with indicated antibodies (*top panel*). The relative ratios (phospho-EGFR, phospho-ERK, and phospho-AKT versus EGFR, ERK, and AKT, respectively) were statistically analyzed (*bottom panel, n* = 3 individual experiments). (**b**) The interaction between integrin α5 and EGFR was increased in the S3–5,11 cells. The indicated cell extracts were immunoprecipitated (*IP*) with anti-GFP agarose, followed by anti-EGFR and α5 antibodies for WB (*top panels*). The whole cell extracts were also subjected to WB using indicated antibodies where α-tubulin was used to check equal loading (*bottom panels*; as an input). All values are reported as the means ± S.E. (*error bars*), Student’s *t*-test; ***p < 0.001.

Given the inhibitory effects of integrin α5 on cell growth via integrin α5-EGFR complex formation^11^, we wondered whether the S3–5,11 mutant could also mimic the WT in its inhibition of the EGFR pathway. As shown in Fig. 2a, the aberrant expression levels of phosphorylated EGFR (p-EGFR) and its downstream molecules ERK and AKT were completely revived in the S3–5,11-CHO-B2/EGFR cells, compared with those in WT or S3–5,10–14 cells. Furthermore, the association between EGFR and integrin α5 was greatly increased in the S3–5,11 cells, which was comparable to those in the WT or S3–5,10–14 cells (Fig. 2b). These results clearly demonstrated that the S3–5,11 mutant and WT integrin α5 have comparable effects on EGFR signaling.

**Figure 3:**
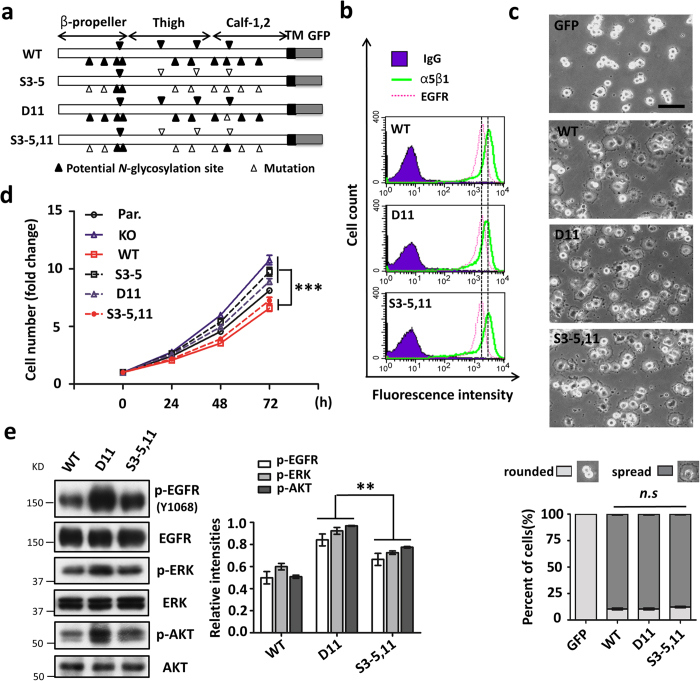
The site-11 *N*-glycosylation of integrin α5 plays a key role in mediating its growth-inhibitory function in HeLa cells. (**a**) Schematic diagram of a potential *N*-glycosylation mutational integrin α5 subunit (WT; S3–5; D11; and, S3–5,11). (**b**,**c**) Comparison of expression levels of α5β1 and EGFR on the cell surface (**b**) and cell spreading (**c**) among the WT; D11; and S3–5,11 cells. The stable rescued-HeLa cell lines were established as described under the “Methods” section. The expression levels of both α5β1 and EGFR on the cell surface were analyzed via flow cytometry (**b**). The cell-spreading abilities of these cells were analyzed, as described in Fig. 1c (*n* = 3 individual experiments). The percentages of rounded and spread cells were statistically analyzed (**c**, *bottom panel, n* = 9). *Scale bar*, 120 μm. (**d**) The site-11 *N*-glycosylation suppressed cell growth. The abilities for cell growth were compared among the parent (Par.); integrin α5-knock-out (KO); WT; S3–5; D11; and S3–5,11 HeLa cells, as described in Fig. 1d (*n* = 3 individual experiments). (**e**) The expression levels of phospho-EGFR and its downstream signaling were significantly increased in the D11, but not in the S3–5,11 cells compared with the WT. *Left panel*, WB pattern; *right panel*, quantitative analysis (*n* = 3 individual experiments). All values are reported as the means ± S.E. (*error bars*), Student’s *t*-test; n.s, not significant (*p* > *0.05*); **p < 0.01; ***p < 0.001.

Moreover, we selected the HeLa cells that express relatively high levels of endogenous integrin α5 and EGFR, in order to further assess whether the phenomenon described above is common to other mammalian cells. The previously established α5-knock-out (KO) HeLa cells^11^ were respectively reconstituted by either the S3–5,11 or D11 α5 mutant via deletion of the *N*-glycosylation of site-11 only through replacing the Asn712 with Asp (Fig. 3a). Therefore, the significance of the potential site-11 *N*-glycosylation of α5 was further confirmed by using the S3–5,11 and D11 HeLa cells. By comparison with the WT cells, both the S3–5,11 and D11 mutant cells exhibited similar expression levels of EGFR and α5β1 on cell surfaces analyzed by flow cytometry (Fig. 3b), as well as comparable abilities for cell spreading on FN (Fig. 3c). Consistent with the data obtained from CHO-B2/EGFR cells, the inhibitory effect on cell proliferation was also observed in the S3–5,11-HeLa cells, when compared with the S3–5 or KO cells (Fig. 3d). It is noteworthy that the inhibitory effect of α5 on cell growth was largely cancelled in the D11 cells, as exhibited by the S3–5 mutants (Fig. 3d). Furthermore, the phosphorylation levels of EGFR and the activations of ERK and AKT were significantly increased in the D11 cells (Fig. 3e), compared with those in the WT or S3–5,11 cells. These results clearly showed that the function of WT and S3–5 mutant integrin α5 on the EGFR of cell proliferation could be duplicated by the S3–5,11 and D11 mutants, respectively, which further highlighted the importance of site-11 *N*-glycosylation in the regulation of EGFR signaling. Therefore, we employed the S3–5,11 and D11 mutant HeLa cells in subsequent experiments to further clarify the underlying molecular mechanism.

### Site-11 *N*-glycosylation inhibits EGFR dimerization in HeLa cells

**Figure 4:**
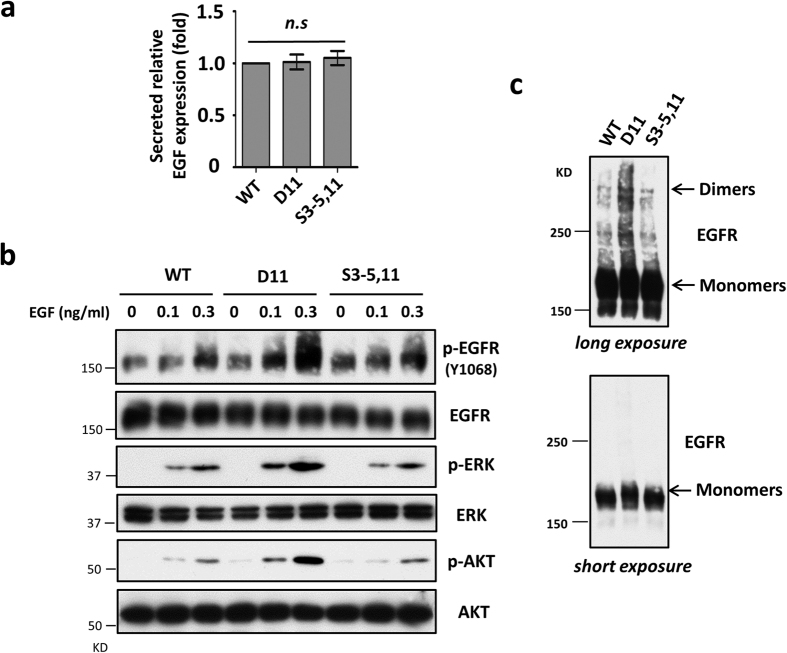
The site-11 *N*-glycosylation of integrin α5 inhibits EGFR dimerization and responses upon EGF stimulation in HeLa cells. (**a**) The WT; S3–5,11; and D11 cells exhibited secretion ability identical to that of EGF. EGF concentrations in the condition media were analyzed by EGF ELISA as described under the “Methods” section. The EGF concentrations in the D11 and S3–5,11 mutants were normalized to WT cells (*n* = 3 individual experiments). All values are the means ± S.E. (*error bars*), Student’s *t*-test; n.s, not significant (*p* > 0.05). (**b**) The responses to EGF were decreased in S3–5,11, but not D11 cells, compared with WT. After starvation for 24 h, cells were treated with EGF at the indicated concentrations for 5 min. WB analysis was performed with indicated antibodies. (**c**) The EGFR dimerization abilities were decreased in the WT and S3–5,11 cells, compared with the D11 cells. After starvation for 24 h, cells were treated with EGF (0.1 ng/ml) for 5 min and subsequently cross-linked using 5 mM Sulfo-EGS as a chemical cross-linker on ice for 2 h and stopped with 10 mM Tris for 15 min. Cell lysates from those cells were subjected to WB to detect EGFR monomers and dimers under long exposure (*top panels*) and short exposure (*bottom panels*) conditions, respectively. EGFR monomers were used as a loading control.

As described above, the site-11 *N*-glycosylation of integrin α5 plays a crucial role in the inhibition of EGFR signaling. Next, we explored the underlying mechanisms involved in this phenomenon. Considering that the expression levels of EGFR (or EGF) and the response ability to EGF are important controlling factors for EGFR activation^33^, we next asked whether the secretion ability of EGF in D11 cells differed from others, since the site-11 *N*-glycosylation did not affect the expression level of EGFR on the cell surface, as shown in Fig. 3b. The ELISA data showed similar expression levels of EGF secreted into the condition media among the WT, D11 and S3–5,11 cells (Fig. 4a), indicating that the increased EGFR signaling in D11 cells was not due to the EGF secretion step, so we assessed the effects of EGF in these cells. In contrast to WT or S3–5,11, the responses to EGF stimulation, as reflected by the expression levels of p-EGFR, p-ERK and p-AKT, were significantly increased in the D11 cells in a dose-dependent manner (Fig. 4b). These results suggest that EGFR activation can be suppressed in the presence of the *N*-glycosylation of site-11 in α5.

Given the evidence that the dimerization of EGFR is an initial and essential event in EGF-induced signal transduction^34^, we compared EGFR dimerization in these three cell lines to further study the mechanism involved in site-11 *N*-glycosylation-mediated EGFR inhibition. As shown in Fig. 4c, upon EGF treatment, more EGFR dimers were visualized in D11 cells compared with either the WT or S3–5,11 cells. Collectively, these data suggested that the site-11 *N*-glycosylation of integrin α5 suppresses the dimerization of EGFR, resulting in an inhibition of EGFR-mediated signaling.

### Loss of the *N*-glycosylation of site-11 switches the EGFR complex formation from α5β1 to α6β4

**Figure 5:**
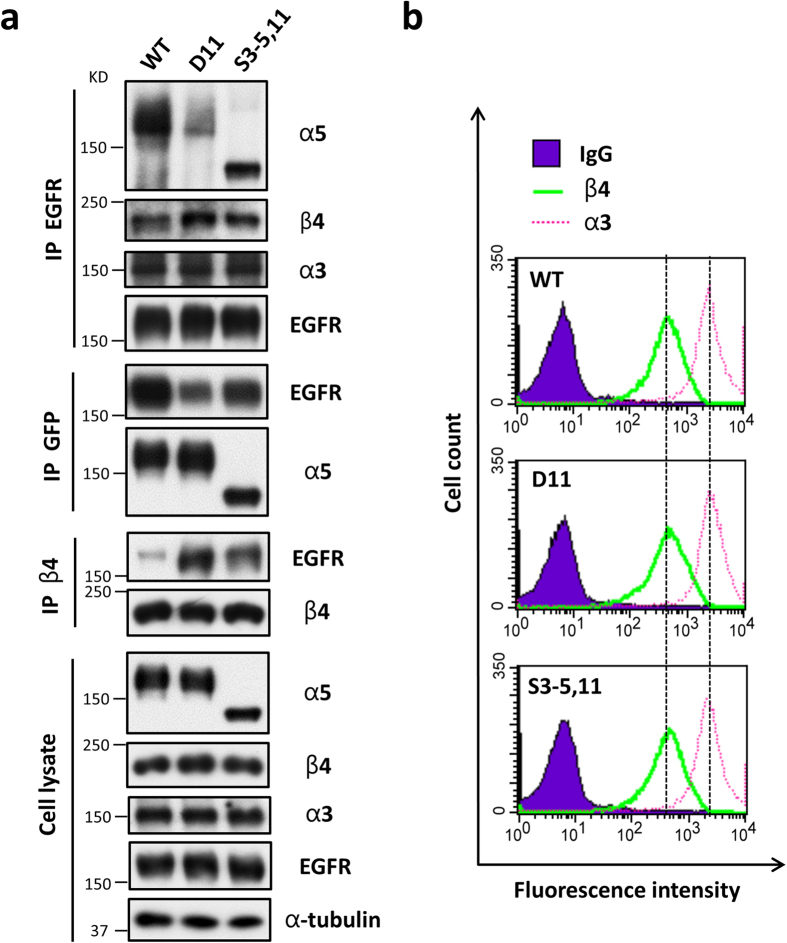
The site-11 *N*-glycosylation of integrin α5 inhibits the EGFR-integrin β4 complex formation in HeLa cells. (**a**) Comparison of complex formations between EGFR-α5 and EGFR-β4 among the WT; D11; and S3–5,11 cells. The indicated HeLa cell extracts were IP with anti-EGFR antibody (*top IP panels*), anti-GFP agarose (*middle IP panels*), or anti-integrin β4 antibody (*bottom IP panels*), and then subjected to WB, reciprocally followed by anti-integrin α5, β4, α3, or EGFR antibodies for detection. The whole cell extracts were also subjected to WB as an “input” using the indicated antibodies (*bottom panels*). (**b**) The WT; D11; and S3–5,11 cells exhibit the same expression level of integrin β4 and α3 on the cell surface. The expression levels of both integrins β4 and α3 were analyzed by flow cytometry. The IgG was used as a control.

Next, we wondered how the site-11 *N*-glycosylation participates in the inhibition of EGFR signaling. Considering the notion that integrin α5-mediated EGFR-α5 complex formation can restrict EGFR signaling^11^, we checked the effects of the site-11 *N*-glycosylation of EGFR-α5 complex formation via immunoprecipitation experimentation. Reciprocal immunoprecipitates with anti-GFP agarose or anti-EGFR antibody showed that the interaction between integrin α5 and EGFR was significantly decreased in D11 cells, by comparison with the WT or S3–5,11 cells (Fig. 5a), indicating that site-11 *N*-glycosylation plays a key role in EGFR-integrin α5β1 complex formation.

Given the fact that the crosstalk between other integrins and EGFR is also important for EGFR signaling^4^,^5^,^35^,^36^, we also detected an association between EGFR and other integrins, including integrin β4 and α3, which are highly expressed in HeLa cells. We initially detected the expression levels of integrin β4 and α3 in these cell lines. As shown in Fig. 5a,b, these cell lines exhibited the same expression levels of integrin β4 and α3 in the whole-cell lysates (Fig. 5a, lower panels) and on the cell surface, as analyzed by flow cytometry (Fig. 5b), respectively, suggesting that the site-11 *N*-glycosylation had no effect on the expressions of integrin β4 and α3. Interestingly, the interaction between EGFR and integrin β4, but not α3, was significantly increased in the D11 cells compared with that in WT or S3–5,11 cells, which was opposite to the patterns observed in the case of α5 (Fig. 5a). These results indicate that the *N*-glycosylation of site-11 in integrin α5 mediated its association with EGFR, which may inhibit the EGFR-integrin β4 complex formation that is known to promote EGFR-mediated signaling^36^,^37^.

### Integrin α5 and β4 competitively associate with EGFR to regulate its signaling in controlling cell proliferation via the site-11 *N*-glycosylation of α5

**Figure 6:**
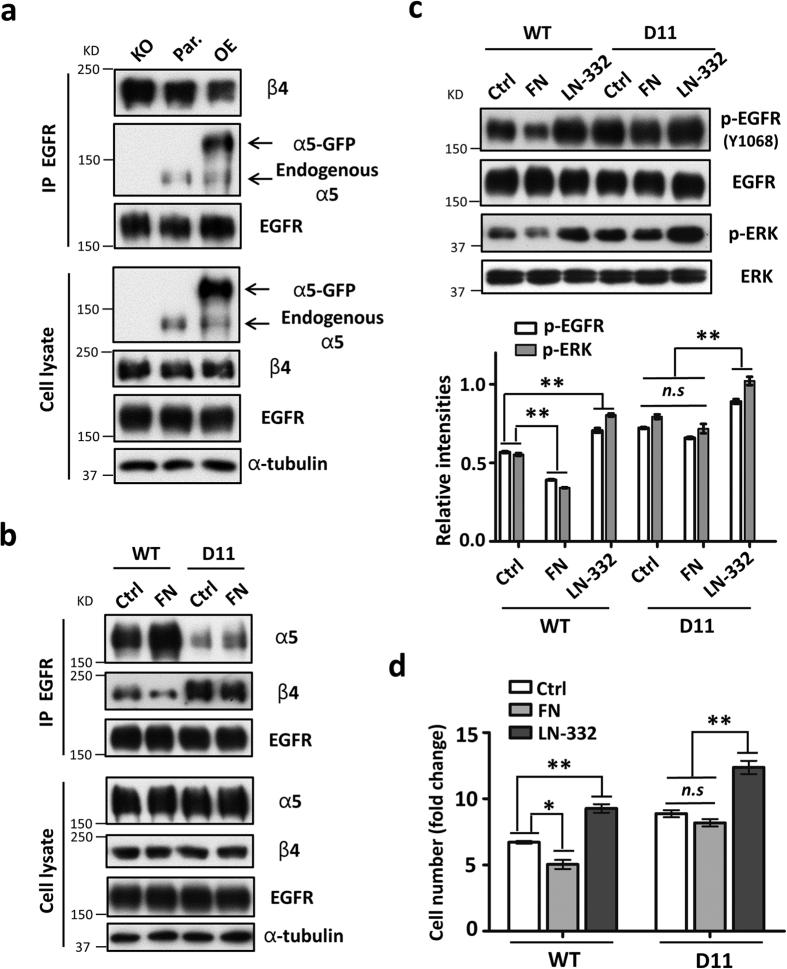
Integrin α5 and β4 competitively associated with EGFR and regulated its-related cellular signaling via site-11 *N*-glycosylation of α5. (**a**) The competitive relationship between integrins α5 and β4 to interact with EGFR in HeLa cells. The integrin α5 (GFP-tagged)-overexpressed HeLa stable cell line (OE) was established as described under the “Methods” section. The extracts from integrin α5-knock-out (KO), parent (Par.), and OE HeLa cells were IP with anti-EGFR antibody followed by anti-integrin α5, β4, and EGFR antibodies for WB (*top panels*). (**b**) The interaction patterns between EGFR and integrins β4 or α5 in the WT and D11 HeLa cells spread on FN. The indicated cells were cultured on dishes pre-coated with or without 10 μg/ml FN for 24 h. The resultant cell lysates (as an input; *bottom panels*) were directly blotted with indicated antibodies or IP with anti-EGFR antibody (*top panels*) and then blotted with anti-integrin α5, β4, and EGFR antibodies. (**c**,**d**) Comparison of the phospho-EGFR and phopho-ERK expressions (**c**) and cell proliferation abilities (**d**) between the WT and D11 cells spread on FN or laminin-332 (LN-332). The WT and D11 cells were cultured on dishes pre-coated with or without 10 μg/ml FN or 1 μg/ml LN-332 for 24 h. The resultant cell lysates were subjected to WB with indicated antibodies (c*, top panels*), the relative ratios were statistically analyzed (c*, bottom panels, n* = 3 individual experiments). The analysis of cell growth was performed as described in the legend to Fig.1d, the related cell numbers at 72 h were counted and statistically analyzed (d*, n* = 3 individual experiments). All values are reported as the means ± S.E. (*error bars*), Student’s *t*-test; n.s, not significant (*p* > 0.05); *p < 0.05; **p < 0.01.

Based on the opposite complex formation patterns of EGFR-α5 and EGFR-β4 in the mutants described above, we hypothesized that the binding of EGFR to integrin β4 may be suppressed by the competition from α5 expression. To test this idea, we initially overexpressed the GFP-tagged integrin α5 in the parent HeLa cells and then detected the effect on the EGFR-integrin β4 complex formation. As shown in Fig. 6a, the interaction between integrin β4 and EGFR was greatly increased in the α5-KO cells, but was decreased in the α5 overexpression cells, which confirmed our hypothesis. Consistently, when cells proliferated on FN-coated dishes in the WT cells, the interaction between α5 and EGFR was increased, but the EGFR-integrin β4 association was accordingly decreased (Fig. 6b). In contrast with the changes observed in the WT cells, the associations of EGFR-integrin α5 and EGFR-integrin β4 were, to some extent, increased and decreased, respectively, in the D11 cells upon FN treatment (Fig. 6b). Importantly, the association between EGFR-integrin β4 was significantly increased in the D11 cells, compared with the WT. These data indicate that integrins α5 and β4 competitively associate with EGFR via the *N*-glycosylation of site-11 in α5.

We then investigated whether the EGFR signaling for cell proliferation was also regulated in the interplay between the EGFR-integrin α5 and EGFR-β4 complex formation. The EGFR-related cellular signaling and cell proliferation abilities were examined in WT and D11 cells grown on dishes coated with either FN or laminin-332 (LN-332), which is a specific ligand for integrin α6β4. In the WT cells, the phosphorylation levels of EGFR and ERK (Fig. 6c), as well as cell proliferation (Fig. 6d), were significantly decreased upon FN treatment, but these were significantly increased under LN-332 treatment. Compared with WT, the D11 cells expressed higher levels of EGFR signaling (Fig. 6c) and cell proliferation (Fig. 6d) upon their respective treatments. Of note, the D11 cells cultured on FN-coated dishes, showed no significant decreases in either EGFR signaling or cell proliferation, whiles the cells cultured on LN-332-coated dishes still exhibited significant increases in both EGFR signaling and cell proliferation, compared with those in the cells without coating. These data strongly suggested that site-11 *N*-glycosylation plays a crucial role in the EGFR-mediated cellular signaling in controlling cell proliferation. Collectively, these results clearly demonstrated that the *N*-glycosylation of site-11 on integrin α5 is the most important factor for the regulation of EGFR signaling through its complex formation with integrin α5 or β4.

### The importance of site-11 *N*-glycan structures in the EGFR-α5 complex formation

**Figure 7:**
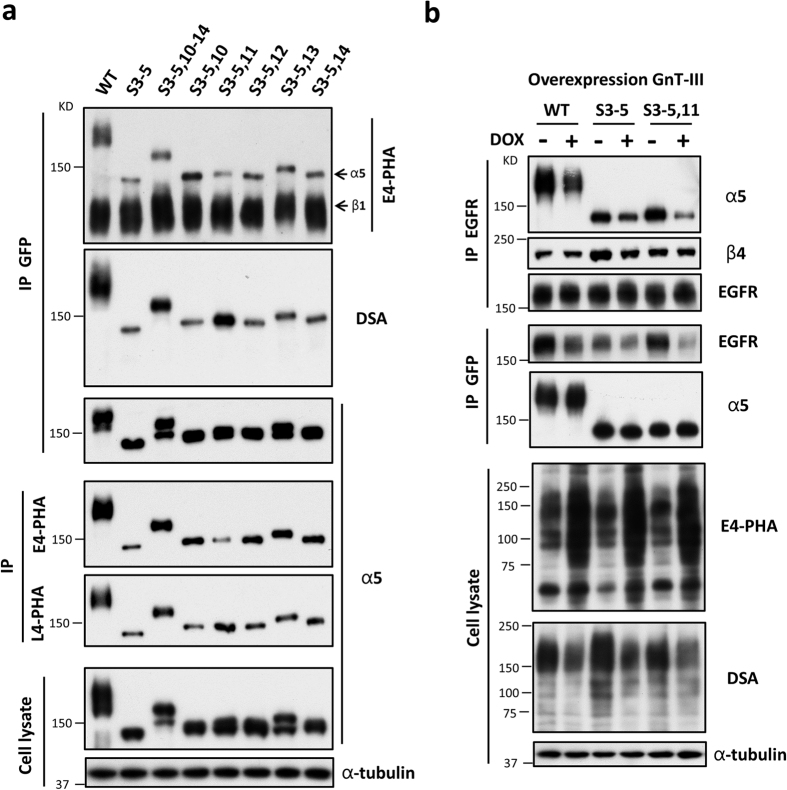
Comparison of *N*-glycosylation patterns on various unglycosylated α5 mutants by lectin blots and the effects of GnT-III overexpression on EGFR-α5 complex formation. (**a**) The cell lysates from WT and unglycosylated mutant CHO-B2/EGFR cells (as an input; *bottom panels*) were directly blotted with indicated antibodies or IP with anti-GFP agarose (*top panels*), E4-PHA-agarose or L4-PHA-agarose (*middle panels*), and then subjected to WB, reciprocally followed by E4-PHA and DSA lectins or anti-integrin α5 antibody for detection. (**b**) Comparison the effects of GnT-III on EGFR-α5 and EGFR-β4 complex formation in WT, S3–5, and S3–5, 11 HeLa cells. The doxycycline (DOX)-inducible GnT-III overexpression stable cell lines were established as described under the “Methods” section. The indicated cells were cultured in the presence (+) or absence (−) with 1 μg/ml doxycycline for 72 h, and the cell extracts were IP with anti-EGFR antibody (*top panels*) or anti-GFP agarose (*middle panels*), and then subjected to WB, reciprocally followed by anti-integrin α5, β4, or EGFR antibodies for detection. The whole cell extracts were also subjected to WB as an “input” using anti-α-tubulin antibody or E4-PHA and DSA lectins (*bottom panels*).

The question regarding *N*-glycosylation of site-11 on integrin α5 is why this is the most important for its-mediated EGFR signaling of cell proliferation. We considered whether the structure of site-11 *N*-glycan is different from other sites. Given the fact that GnT-III is an important glycosyltransferase that catalyzes the addition of the bisecting GlcNAc on *N*-glycan and plays critical roles in determining the structure of *N*-glycans since the bisecting GlcNAc suppresses further processing and elongation of *N*-glycans to form branching structures^38^, we compared the bisecting GlcNAc structure patterns on S3–5,10; S3–5,11; S3–5,12; S3–5,13; and S3–5,14 α5 mutants. These α5 mutants were immunoprecipitated with anti-GFP antibody and then *N*-glycans were detected using E4-PHA and DSA lectin, which specifically recognizes bisecting GlcNAc and branched *N*-glycans, respectively. Interestingly, the E4-PHA reactivities of the α5 subunits (the upper bands), but not the β1 subunit (the lower bands), were much weaker in S3–5,11 cells, as with the S3–5 cells, than those in other mutant cells (Fig. 7a). Conversely, the stronger reactivities of DSA on α5 were observed in the S3–5,11 cells (Fig. 7a). It is worth mentioning that although we detected no reactivity of DSA on the β1 subunit, which is consistent with previous study^39^, this may nonetheless suggest other important information. Consistently, reciprocal immunoprecipitates with E4-PHA agarose and L4-PHA agarose, which selectively recognizes β1,6-GlcNAc branching structures catalyzed by GnT-V, showed that the expression levels of GnT-III-modified- and GnT-V-modified-α5 were significantly decreased and increased in S3–5,11 cells, respectively (Fig. 7a). These results indicate that the site-11 *N*-glycan of α5 carries fewer bisecting GlcNAc structures, however, more branching structures compared with the other four *N*-glycosylation sites on the calf domain.

Therefore, we wondered whether an increase in the bisecting GlcNAc on the site-11 *N*-glycan of α5 decreases the EGFR-integrins complex formation. The GnT-III-overexpressed WT, S3–5, and S3–5,11 HeLa cells were established. The branching structures, as reflected by DSA lectin blot, were suppressed in the GnT-III-overexpressed cells (Fig. 7b, the lower panels), and this further highlights the competitive relationship between GnT-III and GnT-V. As expected, the reciprocal immunoprecipitation showed that the interactions of EGFR and α5 were decreased in both the GnT-III-overexpressed WT and S3–5,11 cells (Fig. 7b, the upper panels). The association of EGFR and α5 showed a marginal decrease after overexpression of GnT-III in S3–5 cells, which may suggest that other factors such as *N*-glycans of EGFR are also involved in the complex formation. It is noteworthy that the EGFR-β4 complex formation was not changed in the WT or S3–5,11 cells, but were significantly decreased in S3–5 cells after GnT-III overexpression (Fig. 7b). One explanation for these observations is that the EGFR-β4 complex formation can also be suppressed by bisecting GlcNAc structures^22^, which may neutralize the competitive role between α5 and β4 to associate with EGFR as described above (Fig. 6), particularly in the WT and S3–5,11 cells. Taken together, these results strongly suggest that the site-11 *N*-glycan of α5 carries many more branching structures, which may promote the EGFR-α5 complex formation and suppress EGFR signaling for cell proliferation.

## Discussion

**Figure 8:**
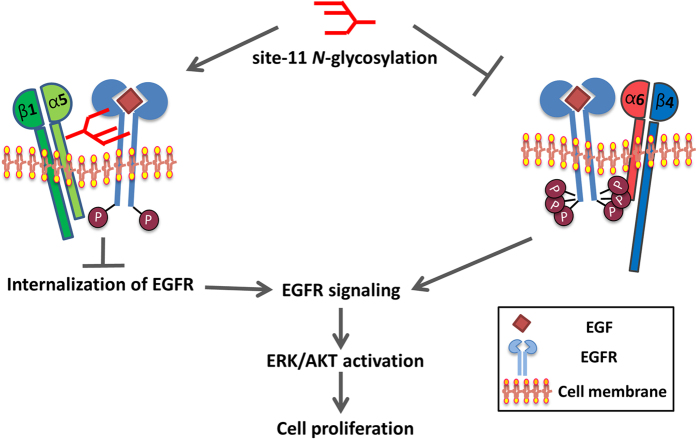
The proposed mechanism for the regulation of EGFR signaling and cell proliferation by the site-11 *N*-glycosylation of integrin α5. The site-11 *N*-glycosylation of integrin α5, which contains abundant branching structures, can serve as a “switch”, which can turn on the EGFR-integrin α5β1 complex formation to restrict the EGFR internalization as described previously^11^, resulting in an inhibition of EGFR-related cellular signaling for cell proliferation (left panel). Meanwhile, the *N*-glycosylation can also switch off the EGFR-integrin α6β4 complex formation due to a mutual competition as described above, which is well known to lead to an activation of EGFR and its downstream signaling (right panel).

In the present study, we intensively studied the effect that *N*-glycosylation of the calf domain in integrin α5 subunit exerted on EGFR signaling in controlling cell proliferation. We found that *N*-glycan of site-11, which contains abundant branching structures, is the most important aspect of its inhibitory effect on EGFR activation. The molecular mechanisms for the inhibition can be mainly ascribed to two aspects. One is that site-11 *N*-glycosylation promoted the interaction of integrin α5β1-EGFR, which may interfere with EGFR endocytosis, as described previously^11^; the other is that this *N*-glycosylation suppressed the EGFR complex formation with integrin α6β4, which is a novel regulatory mechanism for cellular signaling (Fig. 8).

Integrins are thought to crosstalk with EGFR, but an extensive amount of research has been focused on the role of individual integrin subunits and on the results of the activation/inactivation of the EGFR signaling. Previously, we identified integrin α5 as a tumor suppressor via the negative regulation of EGFR signaling^11^. In the present study, we expanded on these observations and found that integrin α6β4 is involved in the inhibitory effect of α5 on cell proliferation, because integrin α5β1 and α6β4 exhibited a mutual competition in the EGFR signaling for cell proliferation. In fact, integrin α6β4 is widely thought to amplify RTK signaling, including EGFR and ErbB2, either via direct promotion of the Src family kinase-dependent phosphorylation of the P loop in the catalytic site of the RTK and RTK-bound substrates such as Gab1^14^,^40^, or by indirect stimulation of the autocrine RTK pathways^7^. Consistently, our results also showed that the EGFR-related cellular signaling and cell proliferation were enhanced with increases in the EGFR-β4 complex formation in the null site-11 *N*-glycosylation mutant. Although the interaction patterns between integrin α3 and EGFR showed no changes among those mutants, we could not exclude the possibility of EGFR signaling from other integrins such as α1β1^19^ and αvβ3^41^. Similar to the competition between integrins α5β1 and α6β4, the functional competition between integrins αMβ2 and α5β1 or integrins αvβ3 and α5β1 was also observed in controlling either leukocyte migration^42^ or trafficking^43^, respectively. In addition, the interplay between integrins and tetraspanins is known to play a crucial role in cell physiology^44^. All these data indicate that the functions of multiple integrins on EGFR are not redundant, and it highlights the importance of the cooperation between individual integrins in cell biology.

It is worth mentioning that integrin α5β1 exerts controversial effects both on EGFR regulation^11^,^13^ and cell proliferation^12^ ranging from stimulatory to inhibitory in different cell types, which is similar to the effects of integrin α6β4 on cell growth^14^,^15^. The information concerning the competition between α5β1 and α6β4 in the way they bind to EGFR, and how that might constitute an overwhelming effect may be one possible explaination for these controversies. In agreement with this hypothesis, our previous data showed that in the MDA-MB-231 and HeLa cells, which express relatively low and high levels of endogenous integrin α6β4, respectively, the inhibition effect of integrin α5β1 on EGFR was attenuated in MDA-MB-231 cells compared with HeLa cells^11^. Although additional analyses is required to fully address the roles of integrin α5β1 in EGFR inactivation in different cells, our data constitute a reasonable argument that the α5β1-mediated inhibition of EGFR is at least partially dependent on α6β4.

Of note, we further expanded our previous observations on the *N*-glycosylation of integrin α5^11^,^32^ and identified the site-11 *N*-glycosylation of the calf domain in α5 as the most important development in the interplay between integrin α5β1 and α6β4 binding with EGFR, which highlights the importance of the individual *N*-glycosylation of α5. Similarly, the importance of individual *N*-glycosylation has also been addressed in other integrin subunits such as β1^45^ or α3^46^. In addition, a recent study demonstrated how *N*-glycosites and site-specific glycoforms of some secreted proteins in drug-resistant gastric cells are distinctly different from those in the parental versions^47^, which indirectly further highlighted the significance of individual *N*-glycosylations. Considering these results, it is reasonable to speculate that an individual *N*-glycosylation site of glycoproteins may exhibit unique biological functions.

The next issue concerned the question of how *N*-glycosylation mediates the α5-EGFR association. We previously demonstrated that the *N*-glycosylation of integrin α5 is important for the α5-ganglioside GM1-EGFR complex formation and its localization in lipid rafts^11^, indicating the significance of the gangliosides in their interaction. Similar to integrin α5, we previously also identified that *N*-glycosylation of integrin β4 is essential for the EGFR-β4 complex formation and localization in lipid rafts^48^. Additionally, gangliosides GM3 and GD3, can interact with the *N*-glycans of EGFR, the so-called cis-carbohydrate to carbohydrate interaction^49^. Therefore, it is reasonable to speculate that some glycolipids may act as a “linker” in the lipid raft for interaction between the *N*-glycosylation of α5 and the *N*-glycan from EGFR. Considering the complexity of the lipid rafts, other compositions such as cholesterol and galectins also can be factors linking α5 and EGFR. In fact, galectin-3 could mediate the association between integrin β4 and EGFR in a GnT-III-dependent manner^22^. Consistently, here we identified that introduction of the bisecting GlcNAc structure modified by GnT-III suppressed the α5-EGFR complex formation, which further indicates that the galectin-3 may also mediate the EGFR-α5 association. Taken together, these data provide evidence for the indirect binding of integrins and EGFR in lipid rafts via specific *N*-glycan structures of integrins. Therefore, it is plausible that the localization patterns of the α5-EGFR and β4-EGFR complexes in lipid rafts may also be controlled by site-11 *N*-glycosylation. In addition to these indirect interactions, we could not exclude the possibility that an unknown lectin-like domain may exist on EGFR, since the lectin domain on integrin αMβ2 is known to play an important role in its association with GlcNAc on the non-reducing terminus of the sugar chains on platelets^50^.

Finally, it is important to further understand why site-11 *N*-glycosylation is the most suitable for the integrin α5-EGFR complex formation of the five putative *N*-glycosylation sites on the calf domain. Although the underlying molecular mechanisms remain unclear, we feel justified in making three speculations based on our observations in the present study. First, the site-11 *N*-glycosylation may localize at a key functional region of α5, which can regulate integrin activation. In fact, introducing an *N*-glycosylation site into integrin β3 or β1 is known to open the hybrid-I-like domain interface of either αvβ3 or α5β1, and increase the ligand-binding affinity^51^. In addition, based on molecular modeling, the *N*-glycans surrounding the RGD-binding pocket on integrin α5 are believed to be essential for its binding to FN^52^, which highlights the importance of the *N*-glycosylation of integrins. Second, the site-11 *N*-glycosylation of α5 may exhibit an appropriate distance to interact with other molecules such as gangliosides GM1 and GM3, which also contributes to the α5-EGFR complex formation described above. Third, the site-11 *N*-glycosylation may exhibit a unique glycan epitope that promotes α5-EGFR complex formation, such as the abundant branching structures observed on site-11 *N*-glycan in this study. Similarly. we previously found that the site-4 *N*-glycan on integrin α5 was specifically modified by GnT-III, which plays a key role in its mediation of cell spreading and migration^39^. Furthermore, the site-heterogeneity of *N*-glycans has also been observed on several other proteins, such as immunoglobulin family proteins^53^. Collectively, these results suggest that both the position and structure of *N*-glycan may play a crucial role in controlling its mediated biological functions. Further studies are required to confirm these ideas.

In summary, this study was particularly focused on the *N*-glycosylations on the calf domain of the integrin α5 subunit. One *N*-glycosylation of site-11 was identified as an “on-and-off” switch that controls EGFR binding to integrin α5β1 and α6β4, which may provide a new perspective on the interplay between integrins and GFRs. In addition, we also highlighted the suppressive effects of GnT-III on regulating the site-11 *N*-glycan-mediated EGFR-α5 complex formation, which may be a plausible molecular mechanism involved in GnT-III-mediated tumorigenesis in several tumors such as human hepatomas, glioma, and ovarian cancers^54^,^55^,^56^.

## Methods

### Antibodies and reagents

The experiments were performed using the following antibodies: monoclonal antibodies (mAbs) against integrin α5 (610634) and integrin β4 (611233) were obtained from BD Biosciences; the mAbs against human integrin α5β1 (MAB1999) and integrin β4 (MAB2059) were from Millipore; the mouse pAbs to EGFR (Sc-120), integrin α3 (Sc-6592), or integrin α3 (Sc-32237) and the rabbit pAbs to integrin β4 (Sc-9090) or EGFR (Sc-03) were from Santa Cruz Biotechnology; rabbit mAbs to EGFR (catalog no. 4267), p-EGFR (catalog no. 3777), ERK1/2 (catalog no. 9102), p-ERK1/2 (catalog no. 4370), AKT (catalog no. 9272), and p-AKT (catalog no. 4060) were from Cell Signaling Technology; and, the mAb against α-tubulin was from Sigma. Biotinylated erythroagglutinating phytohemagglutinin (E4-PHA) and Datura stramonium agglutinin (DSA) lectins were obtained from Seikagaku Kogyo Inc. (Tokyo, Japan). Alexa Fluor^®^ 647 goat anti-mouse IgG was obtained from Invitrogen (Life Technologies). The peroxidase-conjugate goat against mouse, donkey against goat, and goat against rabbit IgG antibodies were obtained from Chemicon, Santa Cruz Biotechnology, and Cell Signaling Technology, respectively. The fibronectin (FN) and laminin-332 (LN-332) were from sigma and Oriental Yeast Co., Ltd. (Tokyo, Japan), respectively; the EGF (AF-100) was from PeproTech; the control mouse IgG1 was from TONBO biosciences; and the Sulfo-EGS Biotin was from Thermo Fisher Scientific. The agarose-conjugated anti-green fluorescent protein (GFP) antibody (RQ2) and the Streptavidin-conjugated agarose were obtained from Medical & Biological Laboratories Co., Ltd. (Nagoya, Japan) and Millipore, respectively. The biotinylated-conjugated erythro-agglutinating phytohemagglutinin (E4-PHA)-agarose (J311) and leukoagglutinating phytohemagglutinin (L4-PHA)-agarose (J312) were obtained from J-OILMILLS (Tokyo, Japan). The Quantikine^®^ Human EGF Immunoassay kit was obtained from R&D Systems.

### Cell lines and cell culture

The 293T (for the lentivirus production) and HeLa cell lines were provided by the RIKEN cell bank (Japan). The stable EGFR overexpressed CHO-B2 cell line (CHO-B2/EGFR) and integrin α5-KO HeLa cells were previously established in our laboratory^11^. The other stable cell lines used in this study were established as mentioned below. All cell lines were maintained at 37 °C in Dulbecco’s modified Eagle’s medium (DMEM), supplemented with 10% fetal bovine serum (FBS), under a humidified atmosphere containing 5% CO_2_, except for the virus production.

### Integrin α5 and GnT-III expression vectors

The vectors of GFP-tagged-WT; S3–5; and S3–5,10–14 integrin α5 were previously established in our laboratory^32^. The mutation vectors (S3–5,10; S3–5,11; S3–5,12; S3–5,13; S3–5,14; and, D11) were constructed using a site-directed mutagenesis kit (Takara Bio) according to the manufacturer’s instructions. The resultant cDNAs were sequenced to confirm the presence of the desired mutations. We used a Gateway^TM^ cloning System kit (Thermo Fisher Scientific) to acquire all the expression vectors. Briefly, the LR clonase enzyme was used to transfer the cDNAs of integrin α5 from the entry vectors into CSII-CMV-Rfa (for rescuing the related-α5 in CHO-B2/EGFR cells, kindly provided by Dr. H. Miyoshi, Riken, Tokyo, Japan) and CSII-EF-Rfa (for rescuing the related-α5 in α5-KO HeLa cells or overexpressing α5 in parent HeLa cells). The doxycycline (DOX)-inducible GnT-III-overexpressing lentiviral vectors (CSIV-TRE-RfA-CMV-KT-GnT-III) was previously established in our laboratory^28^.

### Virus production and infection

The virus production and infection was performed as described previously^11^. In brief, the CSII-CMV-Rfa (or CSII-EF-Rfa)-based integrin α5- and CSIV-TRE-RfA-CMV-KT-GnT-III- lentivirus vectors were cotransfected with pCAG-HIVgp and pCMV-VSV-G-RSV-Rev into 293T cells. After transfection for 48 h, the lentivirus supernatants were collected. For the integrin α5-overexpression stable cell lines establishment, the CHO-B2/EGFR, α5-KO HeLa, or parent HeLa cells were infected with the related CSII-CMV-Rfa or -CSII-EF-Rfa lentivirus. The GFP-positive cells were sorted 3 times using FACSAria II after infection for 72 h. For the GnT-III-overexpression stable cell line establishment, the infected cells were also selected 3 times by the Kusabira Orange marker using FACS Aria II. The stable cell lines were used in subsequent studies. The expression of GnT-III was induced by addition of 1 μg/ml DOX, and the cells cultured under DOX-free medium were used as the control.

### Western blot (WB) and immunoprecipitation (IP)

For WB, the indicated cells were washed with ice-cold PBS and then lysed in the cell lysate (TBS (20 mM Tris-HCl pH 7.4, 150 mM NaCl) containing 1% Triton X-100) with protease and phosphatase inhibitors (Nacalai Tesque, Kyoto, Japan) for 30 min. After centrifugation at 1,000 × g for 10 min, the supernatant was collected and protein concentrations were determined using a Pierce BCA protein assay kit (Thermo Fisher Scientific). The protein lysates were resolved by non-reducing SDS-PAGE for integrin α5 or reducing SDS-PAGE for other proteins. After electrophoresis, the proteins were transferred to a PVDF membrane (Millipore) and detected with indicated primary and secondary antibodies or with biotinylated lectins as indicated and a Vectastain ABC kit (Vector Laboratories) using an Immobilon Western Chemiluminescent HRP Substrate (Millipore), according to the manufacturer’s instructions. For IPs, cells were lysed with 20 mM TBS buffer without detergent by being passed through a 21-G needle, as described previously^57^. Briefly, cells were resuspended in the TBS with protease and phosphatase inhibitors and lysed by being passed through a 21-G needle 30 times. After centrifugation at 1,000 × g for 10 min, the supernatant was collected. The remaining pellet was again syringed and centrifuged, and the second postnuclear supernatant was combined with the first. The supernatants were immunoprecipitated with anti-GFP-agarose, E4-PHA-agarose, L4-PHA-agarose, anti-EGFR (Sc-03), or anti-integrin β4 (Sc-9090) antibody and Ab-Capcher Protein A-R28 agarose (Protenova, Tokushima, Japan) for 1 h at 4 °C with rotation, then the immunoprecipitates were washed twice with TBS and subjected to 6% SDS-PAGE.

### Cell growth analysis

To assay the cell growth curves, the cells (3 × 10^4^ or 1 × 10^4^) were seeded in FN (10 μg/ml) pre-coated 6-cm dishes or LN-332 (1 μg/ml) pre-coated 24-well plates overnight and then serum-starved for 24 h. After starvation, the cells were supplied with DMEM containing 10% FBS. The photos of the same areas on the cultured dishes were taken at the indicated times (0, 24, 48, and 72 h), and the cell numbers were counted and normalized to those at 0 h for statistical analysis.

### Flow cytometry analysis of cells

Flow cytometric analysis was performed as described previously^45^. Briefly, semi-confluent indicated cells were detached from the 10-cm culture dishes and subsequently stained with either the mouse IgG or primary mouse anti-α5β1, anti-EGFR (Sc-120), anti-α3 (Sc-32237), or anti-β4 (MAB2059) antibody for 1 h on ice, followed by incubation with Alexa Fluor^®^ 647 goat anti-mouse IgG for 1 h. During incubation, the cells were mixed gently every 10 min by flicking. After incubation, cells were washed 3 times with ice-cold PBS, and then analyzed using a FACSCalibur flow cytometer (BD Biosciences).

### Cell-spreading assay

The cell-spreading assays were performed as described previously with minor modifications^32^. Briefly, 6-well plates were coated with FN (10 μg/ml) in PBS overnight at 4 °C and then blocked with 1% bovine serum albumin (BSA) in DMEM for 1 h at 37 °C. The indicated cells were detached and suspended in serum-free DMEM with 0.1% BSA at 3 × 10^4^ cells/ml. After replating on the FN-coated dishes for 20 minutes, non-adherent cells were removed by washing with PBS, and the attached cells were fixed with 4% paraformaldehyde in PBS, and representative photos were then taken by phase contrast microscopy. The ratios of the rounded, spread, or elongated cells were statistically analyzed.

### ELISA

The indicated cells were grown on 6-well plates for 24 h and then incubated with 0.5 ml serum-free media. After incubation for 72 h, the media was collected and stored at −80 °C until assay. The concentration of EGF in each group medium was assayed using a Quantikine^®^ Human EGF Immunoassay kit, according to a procedure described by the manufacturer.

### Chemical cross-linking of EGFR

To assay the dimerization of EGFR, cells were prestarved for 24 h and then treated with EGF (0.1 ng/ml) for 5 min. After stimulation, cells were immediately washed twice with ice-cold PBS and subsequently cross-linked with 5 mM Sulfo-EGS dissolved in PBS on ice for 2 h and then stopped using 10 mM Tris for 15 min. Finally, the cells were solubilized with lysis buffer and subjected to 5% SDS-PAGE, as described above, to detect both EGFR monomers and dimers. Dimers were visualized as >300 kD bands, in which monomers served as the loading control.

### Statistical analysis

Results are reported as the mean ± S.E. Statistical analyses were performed using a Student’s *t* test and GraphPad Prism version 5. Statistical significance was defined as *p* < 0.05 (not significant (*n.s*), *p* > 0.05; *p < 0.05; **p < 0.01; ***p < 0.001).

## Additional Information

**How to cite this article**: Hang, Q. *et al. N*-Glycosylation of integrin α5 acts as a switch for EGFR-mediated complex formation of integrin α5β1 to α6β4. *Sci. Rep.* **6**, 33507; doi: 10.1038/srep33507 (2016).
